# Genome Sequence of *Pseudomonas brassicacearum* DF41

**DOI:** 10.1128/genomeA.00390-14

**Published:** 2014-05-08

**Authors:** Peter C. Loewen, Jack Switala, W. G. Dilantha Fernando, Teri de Kievit

**Affiliations:** a Department of Microbiology, University of Manitoba, Winnipeg, Manitoba, Canada; b Department of Plant Science, University of Manitoba, Winnipeg, Manitoba, Canada

## Abstract

*Pseudomonas brassicacearum* DF41, a Gram-negative soil bacterium, is able to suppress the fungal pathogen *Sclerotinia sclerotiorum* through a process known as biological control. Here, we present a 6.8-Mb assembly of its genome, which is the second fully assembled genome of a *P. brassicacearum* strain.

## GENOME ANNOUNCEMENT

*Pseudomonas brassicacearum* strain DF41 is a canola (*Brassica napus* L.) root isolate that exhibits excellent antifungal activity against *Sclerotinia sclerotiorum* (Lib.) de Bary in both greenhouse and field trials (1, 2). The fungal pathogen *S. sclerotiorum* is not only versatile but is economically important due to its ability to infect >400 plant species (3). Canola, an edible oilseed crop with low saturates and high protein in the meal, belongs to the family *Brassicaceae* (*Cruciferae*). In canola, *S. sclerotiorum* causes stem rot, resulting in up to 100% yield loss under conducive conditions. Strain DF41 produces a number of compounds that are believed to contribute to biological control, including protease, hydrogen cyanide, and a novel lipopeptide called sclerosin (2, 4). Sclerosin is essential for antifungal activity, as a sclerosin-deficient mutant, DF41-1278, showed dramatically reduced fungal inhibition (2). The expression of antifungal metabolites in DF41 is governed by a complex regulatory cascade that includes the GacS-GacA two-component regulatory system (2), the PdfRI quorum-sensing (QS) system (5), a QS-associated regulator called RfiA (5), and the stringent response (6).

The genome of *P. brassicacearum* DF41 was sequenced in two stages. The first stage employed data generated using an Illumina MiSeq platform, which were assembled into 298 contigs using a combination of MIRA Assembler version 3.9.3 (7), Velvet version 1.2.08 (8), and the MUMmer version 3.23 (9) package. The second stage to complete the genome utilized a Pacific Biosciences data set generated by Genome Québec, which was assembled using the PacBio SMRT Analysis pipeline version 2.0.1, with 222× coverage to give a single contiguous genome sequence. The contigs from the Illumina data were aligned for confirmation. The sequence was annotated by the National Center for Biotechnology Information (NCBI) Prokaryotic Genomes Annotation Pipeline.

Our previous attempts to assign a species eponym to DF41 had been unsuccessful, but finding the *cpn60* gene in DF41 to be >97% identical to that of *P. brassicacearum* allowed us to designate DF41 as a member of this species. The *P. brassicacearum* DF41 genome consists of 6,652,396 bases, with a G+C content of 60.5%. There are 5,574 putative coding sequences, 65 tRNA genes, and 5 rRNA clusters. In addition, the biosynthetic loci for lipopeptide molecules, hydrogen cyanide, and alkaline protease have been identified, consistent with the exoproducts secreted by this bacterium. A comparison of the genome with the only other completed *P. brassicacearum* genome, that of strain NFM421 (accession no. CP002585.1) (10), using Mauve 2.3.1 (11), revealed only 72% identity and the presence of a very large inversion of almost 2.5 Mb.

### Nucleotide sequence accession number.

The genome sequence of *P. brassicacearum* DF41 was deposited with NCBI GenBank under the accession no. CP007410.1.
